# Supplementary figures and images for: Structural insights into hormone recognition by the human glucose-dependent insulinotropic polypeptide receptor
Source: eLife. 2021 Jul 13;10:e68719. doi: 10.7554/eLife.68719 (PMC8298097; doi:10.7554/eLife.68719)

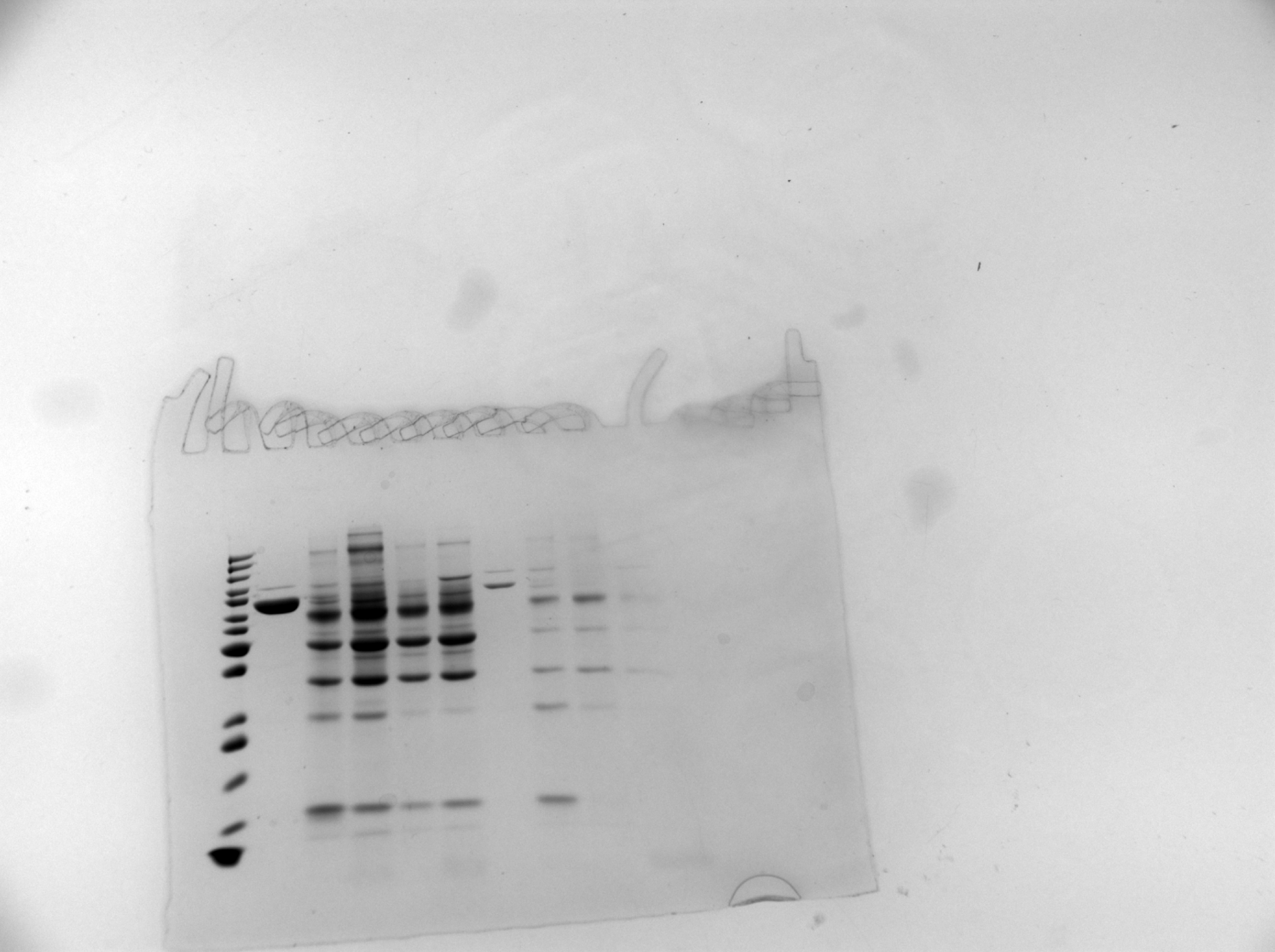

Supplement: Source data 1. [file elife-68719-data1.zip › Source data0712/Figure 1—figure supplement 1D-source data 1.tif]

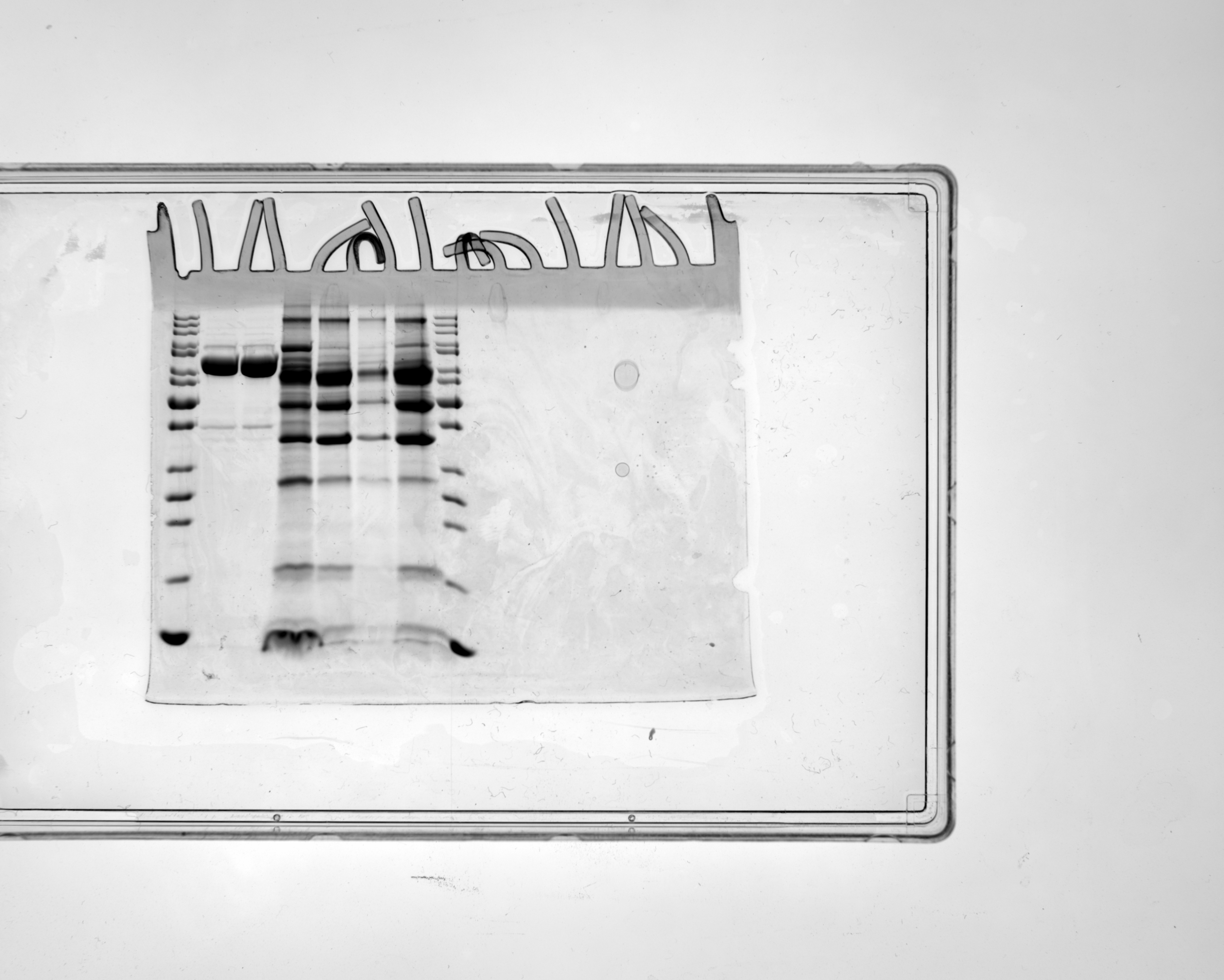

Supplement: Source data 1. [file elife-68719-data1.zip › Source data0712/Figure 1—figure supplement 1F-source data 1.tif]
